# Bit-reproducible parallel phylogenetic tree inference

**DOI:** 10.1093/bioinformatics/btag044

**Published:** 2026-01-29

**Authors:** Christoph Stelz, Lukas Hübner, Alexandros Stamatakis

**Affiliations:** Institute of Theoretical Informatics, Karlsruhe Institute of Technology, Karlsruhe 76131, Germany; Computational Molecular Evolution Group, Heidelberg Institute for Theoretical Studies, Heidelberg 69118, Germany; Institute of Theoretical Informatics, Karlsruhe Institute of Technology, Karlsruhe 76131, Germany; Computational Molecular Evolution Group, Heidelberg Institute for Theoretical Studies, Heidelberg 69118, Germany; Biodiversity Computing Group, Institute of Computer Science, Foundation for Research and Technology - Hellas, Heraklion, Crete GR-730013, Greece; Institute of Theoretical Informatics, Karlsruhe Institute of Technology, Karlsruhe 76131, Germany; Computational Molecular Evolution Group, Heidelberg Institute for Theoretical Studies, Heidelberg 69118, Germany

## Abstract

**Motivation:**

Phylogenetic trees describe the evolutionary history among biological species based on their genomic data. Maximum likelihood (ML) based phylogenetic inference tools search for the tree and evolutionary model that best explain the observed genomic data. Given the independence of likelihood score calculations between different genomic sites, parallel computation is commonly deployed. This is followed by a parallel summation over the per-site scores to obtain the overall likelihood score of the tree. However, basic arithmetic operations on IEEE 754 floating-point numbers, such as addition and multiplication, inherently introduce rounding errors. Consequently, the order by which floating-point operations are executed affects the exact resulting likelihood value since these operations are not associative. Moreover, parallel reduction algorithms in numerical codes re-associate operations as a function of the core count and cluster network topology, inducing different round-off errors. These low-level deviations *can* cause heuristic searches to diverge and induce high-level result discrepancies (e.g. yield topologically distinct phylogenies). This effect has also been observed in multiple scientific fields beyond phylogenetics.

**Results:**

We observe that varying the degree of parallelism results in diverging phylogenetic tree searches (high-level results) for over 31% out of 10 179 empirical datasets. More importantly, 8% of these diverging datasets yield trees that are statistically significantly worse than the best-known ML tree for the dataset (AU-test, *P* < .05). To alleviate this, we develop a variant of the widely used phylogenetic inference tool RAxML-NG, which does yield bit-reproducible results under varying core-counts, with a slowdown of only 0%–12.7% (median 0.8%) on up to 768 cores. For this, we introduce the ReproRed reduction algorithm, which yields bit-identical results under varying core-counts, by maintaining a fixed operation order that is independent of the communication pattern. ReproRed is thus applicable to all associative reduction operations—in contrast to competitors, which are confined to summation. Our ReproRed reduction algorithm only exchanges the theoretical minimum number of messages, overlaps communication with computation, and utilizes fast base-cases for local reductions. ReproRed is able to all-reduce (via a subsequent broadcast) $4.1\times 10^{6}$ operands across 48–768 cores in 19.7–48.61 μs, thereby exhibiting a slowdown of 13%–93% over a non-reproducible all-reduce algorithm. ReproRed outperforms the state-of-the-art reproducible all-reduction algorithm ReproBLAS (offers summation only) beyond 10 000 elements per core. In summary, we re-assess non-reproducibility in parallel phylogenetic inference, present the first bit-reproducible parallel phylogenetic inference tool, as well as introduce a general algorithm and open-source code for conducting reproducible associative parallel reduction operations.

**Availability and implementation:**

ReproRed: https://doi.org/10.5281/zenodo.15004918 (LGPL)—Reproducible RAxML-NG version https://doi.org/10.5281/zenodo.15017407 (GPL)

## 1 Introduction

Next to disseminating results, scientific publications also aim to convince the reader of their validity (Mesirov 2010). While reproducibility is crucial for validating scientific claims (Ivie and Thain 2018), practical attempts to reproduce computational findings frequently fail, e.g. in climate and weather modeling (Demmel and Nguyen 2015), power grid analysis (Villa *et al.* 2009), or phylogenetic tree inference (Darriba *et al.* 2018, Shen *et al.* 2020b).

*Phylogenetic trees* describe the shared evolutionary history among related biological species based on their genomic data. Phylogenetic trees are commonly inferred via Maximum likelihood (ML) based methods that search for the tree and evolutionary model that best explain the observed genomic data (Felsenstein 1981). For this, the currently best-known tree is iteratively being improved by proposing topologically similar trees, and numerically optimizing the branch lengths and evolutionary model to assess if a proposed tree improves upon the score of the current best-known tree. To score a tree, statistical models of evolution are used to compute per-site *likelihood* scores. Given the independence of likelihood calculations between sites, phylogenetic inference tools are commonly parallelized over sites. Subsequently, summing over the per-site scores yields the overall tree score. If a proposed topology has a better likelihood score, we continue optimization from this new tree.

However, this iterative improvement of the best-known tree is known to be numerically unstable under varying CPU core-counts (Darriba *et al.* 2018, Shen *et al.* 2020a). Even if an identical binary is re-executed on the same hardware but using a distinct number of threads/MPI processes, we can already observe diverging tree searches.

We define *bit-reproducibility* as a program producing bit-wise identical results when executed twice on the same input data and under the same settings. While tools such as containerization exist to archive the software environment and experimental settings (Boettiger 2015), hardware is substantially more difficult to archive (Ivie and Thain 2018). In order to reproduce results even without access to the hardware originally used, we thus require algorithms that yield bit-reproducible results on a plethora of hardware environments, including different CPU types and varying parallelization levels.

Note that the bias induced by floating-point related non-reproducibility is fundamentally different from non-reproducibility caused by the stochastic nature of phylogenetic inference. The probability distribution of the inferred trees and signal strength of the MSA are commonly assessed by varying the random seeds of the phylogenetic inference tool over multiple runs, by performing bootstrapping, or via Bayesian inference to infer a posterior probability distribution of trees. In contrast to these controlled and well understood (Sanderson 1989, Hedges 1992, Efron *et al.* 1996) methods, the differences introduced by round-off errors induce an unknown, orthogonal inference. Additional experiments (supplement) indicate that varying the random seed yields substantially more diverse tree topologies than varying the parallelization degree. Varying the random number seed also yields less tree topologies that are significantly (AU-test, *P* < .05) worse than the best-known tree topology. Thus, topological variation as induced by deploying distinct random number seeds is beneficial for properly exploring the tree space; in contrast to topological variation induced by varying parallelization degrees. Whether these numerical deviations affect the biological interpretation of results remains an open question. Further, bit-reproducibility facilitates code debugging and verification (Villa *et al.* 2009, Robey *et al.* 2011, Arteaga *et al.* 2014, Demmel and Nguyen 2015), as well as code extension by other researchers (Schwab *et al.* 2000). We thus advocate for using bit-reproducible implementations of phylogenetic tree searches.

### 1.1 Contribution and outline

We investigate the bit-reproducibility of phylogenetic tree inference across 10 179 empirical datasets under varying core-counts and distinct SIMD parallelization kernels. Our findings qualitatively support those by Shen *et al.* (2020b). We find that 31% of the tree searches diverge; with 8% (out of those 31%) yielding trees that are statistically significantly worse than the best-known tree for the dataset (AU-test, *P* < .05). To address this issue, we introduce a bit-reproducible version of the phylogenetic tree inference tool RAxML-NG (Kozlov *et al.* 2019). This bit-reproducible variant only exhibits a slowdown of 0%–12.7% (median 0.8%) compared to the non-reproducible reference on 18 large empirical datasets when using up to 768 cores.

For this, we introduce the distributed-memory parallel reduction algorithm ReproRed, which enables bit-reproducible reduction operations for arbitrary associative operators. This versatility sets ReproRed apart from competitors that are confined to summation (Section 3). It also allowed us to integrate ReproRed into the open-source MPI-wrapper library KaMPIng. ReproRed only exchanges the theoretical minimum number of messages, overlaps communication with computation, and utilizes a fast base-case for conducting local reductions. ReproRed performs an all-reduction (via a subsequent broadcast) on $4.1\times 10^{6}$ operands across 48–768 cores in 19.7–48.61 μs, thereby yielding in a slowdown of 13%–93% over a non-reproducible all-reduce algorithm and 8%–25% over a non-reproducible Reduce-Bcast algorithm. ReproRed outperforms the state-of-the-art reproducible all-reduction algorithm, ReproBLAS (summation only) for more than 10 000 elements per PE.

The remainder of our article is organized as follows: In Section 2, we provide the necessary background and definitions for the remainder of our work. We discuss related work in Section 3 and detail the methods (Section 4) we utilize to quantify the non-reproducibility of phylogenetic inference (Section 4.1) as well as our bit-reproducible reduction algorithm (Section 4.2). Next, in Section 5, we detail the bit-reproducible variant of the phylogenetic search tool RAxML-NG. In Section 6, we describe our experimental setup, and subsequently present our large-scale study on the reproducibility of phylogenetic tree searches in Section 6.1. Section 6.2 provides an empirical evaluation of ReproRed’s runtime compared to the state-of-the-art bit-reproducible (all-)reduction algorithm ReproBLAS. Following this, we assess the runtime penalty induced to RAxML-NG by using bit-reproducible reduction (Section 6.3), discuss reproducibility in Bayesian phylogenetic inference (Section 7), summarize our findings, and discuss directions of future research (Section 8).

## 2 Preliminaries

### 2.1 Parallel reduction and all-reduction

Given an input array of elements $E=[e_{0},e_{1},\ldots ,e_{n-1}]$, distributed across *p* processing elements (PEs; e.g. processes or threads), we desire to compute $r=e_{0}⊕e_{1}⊕\ldots ⊕e_{n-1}$, where ⊕ denotes a binary, associative operation (e.g. summation or multiplication). In a distributed *reduction*, we return the result *r* to a single PE, while in a distributed *all-reduction*, we return *r* to all PEs. Conceptually, an all-reduction therefore comprises a reduction that is followed by a broadcast. However, this approach results in a latency of $2\cdot {\;\text{log}\;}_{2}(p)$ messages: ${\;\text{log}\;}_{2}(p)$ messages for the reduction phase and ${\;\text{log}\;}_{2}(p)$ messages for the broadcast phase (Sanders *et al.* 2019, ch. 13.2.1). Dedicated all-reduction algorithms, such as recursive doubling which is implemented in MPICH (Balaji and Kimpe, 2013), exhibit a latency of only ${\;\text{log}\;}_{2}(p)$ messages.

KaMPIng (Uhl *et al.* 2024) is a high-level C++ wrapper for MPI, which includes the reduction and all-reduction collective operations. The user can provide arbitrary associative reduction operations to KaMPIng’s reduce and allreduce functions. Note that associativity is a necessary requirement for a *parallel* reduction.

### 2.2 Floating-point math is non-associative

Fundamental arithmetic operations, such as additions or multiplications, on IEEE 754 (IEEE754 2019), floating-point numbers induce rounding errors (Goldberg 1991). Thus, the compiler’s choice of CPU instructions (e.g. Fused-Multiply-and-Add (Intel 2024, ch. 14.5.2)), which is often based on the available CPU type, influences the magnitude and propagation of these rounding errors. In addition, inconsistent floating-point related CPU settings can also cause different rounding errors. Examples include the IEEE 754 rounding mode (Intel 2024, ch. 4.8.4), denormalized floating-point numbers (Intel 2024, ch. 10.2.3.4), floating-point exceptions (Corden and Kreitzer 2018), and x87 register precision (Intel 2024, ch. 8). Henceforth, we therefore assume that the exact same instructions are used in each program execution. This can be achieved, for instance, by archiving the compiled binary or by passing the respective reproducibility flags to the compiler (Corden and Kreitzer 2018).

### 2.3 Non-reproducibility of distributed reduction

**Figure 1:**
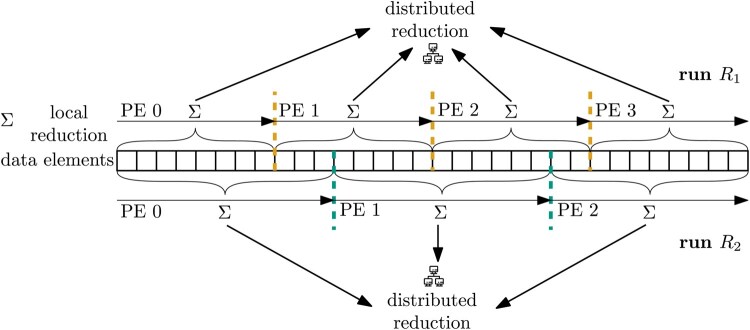
Common distributed-memory parallel reduction algorithms are non-reproducible if the PE-count differs between runs. First, each PE reduces its local elements to a single, intermediate, per-PE result. Next, these intermediate results are exchanged via messages over the network and further reduced into a single element. Here, the PE-count influences the reduction operation order and thus how results are rounded.

In common distributed-memory parallel reduction algorithms, the PE-count influences the operation order and thus, the end-result (Li *et al.* 2023, Siklósi *et al.* 2024) (Fig. 1). This is because the data are often distributed across all PEs in parallel algorithms, with each PE contributing a partial result to the reduction. Changing the parallelization level induces a different data distribution and communication pattern, which in turn alters the order of reduction operations. In addition, topology-aware reduction algorithms, such as in MPICH, adjust the reduction tree topology to accommodate for the heterogeneous connectivity among PEs (Balaji and Kimpe 2013), and thus yield non-reproducible results even under a constant PE-count.

## 3 Related work

Darriba *et al.* (2018) first reported that ML phylogenetic tree searches can diverge due to floating-point inaccuracies. Following this observation, Shen *et al.* (2020b) systematically analysed 3515 single-gene datasets. They observed that variations in the CPU model, SIMD instruction set being used, and the number of threads, yielded different phylogenetic trees for 9%–18% of empirical single-gene datasets. Furthermore, Shen *et al.* observed that 8.6% of the non-reproducible phylogenetic trees found by IQ-TREE and 25.21% of the non-reproducible phylogenetic trees found by RAxML-NG were statistically significantly worse than the respective best-known tree [*P* < .05; AU-test (Shimodaira 2002)].

## 4 Methods

### 4.1 Quantifying the non-reproducibility of phylogenetic tree inference

In Section 6.1, we evaluate the reproducibility of phylogenetic tree inferences on datasets from the TreeBASE (Piel *et al.* 2009) repository. For this, for each dataset, we execute the same RAxML-NG (Kozlov *et al.* 2019) application binary eight times, using identical hardware, search settings, and random seeds. For each of the eight runs we exclusively vary the degree of parallelization. Specifically, we conduct inferences using one to five PEs with AVX2 vectorization, as well as sequential (single core) inferences with SSE3, AVX, AVX2, and auto-vectorization.

We quantify the divergence between the resulting trees using the relative Robinson–Foulds distance (Robinson and Foulds 1981), which corresponds to the proportion of non-trivial bipartitions (i.e. bipartition induced by cutting *inner* branches) that differ between the two phylogenetic trees. The set of non-trivial bipartitions induced by all inner edges fully describe a tree.

When multiple trees exhibit sufficiently similar likelihood scores, there is no compelling reason to favor one over the others. Thus, following Shen *et al.* (2020b), we apply the approximately unbiased (AU) test (Shimodaira 2002) to determine if two topological distinct trees—resulting from inferences under different levels of parallelism—exhibit significantly different likelihood scores (*P* < .05). To prevent erroneous rejection of the correct tree in sets with similar likelihoods (Shimodaira 2002), we consider trees with a log-likelihood difference of less than $10^{-3}$ to not be significantly different.

### 4.2 Bit-reproducible (all-)reduction algorithms

A key ingredient in a parallel bit-reproducible code is a bit-reproducible reduction algorithm. Such (all-)reduction algorithms that yield bit-identical results under varying PE-counts have been studied for both, shared-memory (Villa *et al.* 2009) and distributed-memory (Iakymchuk *et al.* 2015, Chohra *et al.* 2017) systems.

Villa *et al.* (2009) developed a bit-reproducible reduction algorithm on a shared-memory Cray XMT machine and utilized it to sum over floating-point numbers on up to 16 PEs. Their algorithm ensures bit-reproducibility by fixing the reduction operation order independently of the PE-count, thereby supporting arbitrary associative reduction operations. Their *reduction* tree is based on a binomial tree, however, they accumulate k>2 instead of 2 values sequentially at each inner node, which reduces the required tree height. Villa *et al.*’s algorithms are not open-source and are designed for a shared-memory Cray system (which we do not have access to), yielding a comparison to our distributed-memory algorithm infeasible.

For distributed-memory machines, one has to consider explicit communication between PEs as well as scheduling the reduction operations to PEs. Although the MPI-standard encourages bit-reproducible reduction implementations (MPI 2023, ch. 6.9), existing MPI implementations prioritize performance over reproducibility (Balaji and Kimpe 2013). While IntelMPI and MPICH also offer reproducible reduction (Intel 2025, Balaji and Kimpe 2013), they both require an identical PE-count between runs. Further, Balaji and Kimpe (2013) claim that reproducible reduction cannot utilize knowledge about the concrete HPC topology for improving communication efficiency. However, we separate the communication pattern from the computation of the reduction and thereby demonstrate that this is not necessarily the case.

Numerous bit-reproducible parallel *summation* algorithms exist (Arteaga *et al.* 2014, Collange *et al.* 2015, Demmel and Nguyen 2015, Chohra *et al.* 2017, Ahrens *et al.* 2020, Li *et al.* 2023). These algorithms utilize variants of Kahan’s compensated summation (Siklósi *et al.* 2024), which accumulates errors from each pair-wise summation in a separate variable. Thereby these algorithms effectively increase the number of significant bits. Demmel and Nguyen (2013) extend this concept to multiple variables, where each variable successively accumulates smaller parts of the error (Siklósi *et al.* 2024). However, these approaches are summation-specific and do not generalize to other reduction operations. Further, most of these approaches require a specific data representation and have a single target hardware architecture, which complicates code maintenance (Siklósi *et al.* 2024).

The above algorithms have been implemented in three highly-optimized BLAS libraries: ReproBLAS (Ahrens *et al.* 2020), RareBLAS (Chohra *et al.* 2017), and ExBLAS (Iakymchuk *et al.* 2015). However, RareBLAS (Chohra *et al.* 2017) targets parallel shared-memory machines, yielding it inapplicable to our distributed-memory context. Further, Lei *et al.* (2023) show that ReproBLAS is consistently faster than ExBLAS. Consequently, we omit ExBLAS and only consider ReproBLAS as the state-of-the-art implementation for evaluating our ReproRed algorithm.

### 4.3 Further baselines

The *Gather-Bcast* algorithm initially gathers all input data elements on a single root PE, which subsequently reduces all elements in a fixed order, for instance, from left to right. The root PE then broadcasts the reduced result to all other PEs. The MPI-standard recommends this algorithm for obtaining bit-reproducible results under varying PE-counts (MPI 2023, ch 6.9). However, this approach incurs a bottleneck communication volume of p−1 messages and *n* received elements at the root PE, where *p* is the number of PEs, and *n* is the number of input data elements. Further, this algorithm lacks parallelism, resulting in a runtime of O(n). Moreover, the root PE requires sufficient memory to hold *all* input data elements.

We also compare ReproRed against two *non-reproducible* baselines: a PE-local reduction using C++’s std::reduce followed by either IntelMPI’s MPI_Allreduce, or a MPI_Reduce and a subsequent MPI_Bcast. We denote these algorithms as *Allreduce* and *Reduce-Bcast*, respectively. In our experiments, IntelMPI selects the “best” (all-)reduction algorithm by using yet undisclosed heuristics. We attempted to re-configure IntelMPI to use a binomial tree broadcast, binomial tree reduction, and recursive doubling all-reduction by adjusting the respective I_MPI_ADJUST variables. However, this resulted in performance degradation, possibly because of the loss of topology-aware optimizations. We therefore perform all benchmarks under IntelMPI’s default settings.

### 4.4 Operation-agnostic reproducible reduction

**Figure 2:**
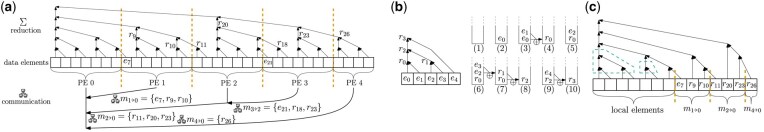
Implementation of an operation-agnostic bit-reproducible parallel reduction in ReproRed. (a) Decoupling the reduction operation order from the communication pattern. ReproRed reduces the data elements along a fixed tree, regardless of the number and topology of the PEs. The *p* PEs communicate via a binomial tree, which requires exchanging p−1 messages (the theoretical minimum). (b) A post-order traversal of a binary tree (left) can be implemented via a stack (right). When we encounter a leaf node during the traversal, we push the respective data element onto the stack. For a non-leaf node, we pop the two topmost elements from the stack, reduce them, and push the result back onto the stack. At the root of the tree, the remaining stack element contains the final result of the post-order traversal. (c) Reduction operations performed on PE 1 in (a). The received messages $m_{i\to j}$ are stored consecutively after the local elements. Note that some received elements already constitute intermediate results of the overall reduction. Further, the PE can reduce most of its local elements (green, dashed boxes) and thereby overlap computation with communication.

The only approach for ensuring bit-reproducibility for arbitrary (associative) reduction operations, is to maintain a fixed reduction operation order (Arteaga *et al.* 2014). To this end, we orchestrate the reduction operations via a binary tree with the input data elements located at its leaves (Fig. 2c). This *pair-wise reduction* also decreases the overall rounding errors (Higham 1993). Further, we orchestrate communication via a binomial tree, which is theoretically optimal for small message sizes (Sanders *et al.* 2019, ch. 13.9), and used, for instance, by MPICH (Balaji and Kimpe 2013). Note that this design implies that PEs can generally not reduce all their local elements into a single intermediate result, but are required to send the intermediate results of multiple subtrees to their parent in the communication tree instead. However, we expect the startup overhead for each message to exceed the cost of transmitting the respective additional data elements.

We implement the post-order traversal of the reduction tree via a stack *S* (Fig. 2b). Again, let $E=(e_{0},e_{1},\ldots ,e_{n-1})$ be the *ordered* sequence of elements to be reduced. In the reduction tree, each *leaf* (in-degree 0) corresponds to an element $e_{i}\in E$. When we encounter a leaf during the post-order traversal, we push the corresponding element onto *S*. We denote this as *addValue*. Conversely, each *non-leaf* node of the reduction tree represents an intermediate result $r_{j}$, which we index in post-order. When we reach a non-leaf node, we *pop* the two topmost elements from *S*, apply the reduction operator ⊕, and *push* the result back onto *S*. We denote this as *reduceTwoValues*. Ultimately, the root of the reduction tree (out-degree 0) contains the final result of the reduction. This will be the sole remaining element on the stack. Therefore, we can implicitly represent the reduction via a sequence of *addValue* and *reduceTwoValues* operations.

To parallelize this traversal—and thus the reduction—we distribute this sequence of *addValue* and *reduceTwoValues* operations across all PEs. For this, we assign each *addValue* operation to the PE that holds the respective data element. Each *reduceTwoValues* operation corresponds to a specific non-leaf node in the reduction tree. This node, including its descendants, induces a subtree in the reduction tree, with a set of (consecutive) input data elements as leaf nodes. Each of these elements resides on a specific PE. The PE set on which the elements of a reduction subtree reside have a joint lowest common ancestor (LCA) in the communication tree. We assign the *reduceTwoValues* operation to this LCA-PE.

We determine this assignment of operations to PEs via a pre-processing step, which we execute once for each data distribution. Thus, when one invokes the distributed reduce operation with concrete values—possibly thousands of times per second (Section 6.3)—each PE already knows all operations it needs to execute *a priori* (Fig. 2c).

During a reduction, each PE initially triggers the asynchronous message-receive to overlap computation with communication. As each PE knows the number of messages and data elements it will receive, it can arrange local and received data at consecutive memory locations. The operations of a PE correspond to pair-wise reductions in a subtree of the overall reduction tree. This subtree has input data elements and intermediate results computed on other PEs as leaves. When reducing this local subtree, we apply a fast base-case for fully local data; e.g. we parallelize summation via manually-optimized AVX2 code (see supplement). If a PE needs to perform a *reduceTwoValues* operation for which one operand has not been received yet, the PE blocks until the respective message exchange has been completed. Upon completing its reductions, the PE sends the computed intermediate results to its communication tree parent. Note that the sending PE’s stack will already contain the elements in the order that is expected by the receiving PE.

The three key optimizations we implement are to overlap computation with communication, a fast base-case for reducing PE-local data elements, and to communicate via a binomial tree of degree four instead of degree 2 (see Supplementary material).

## 5 Reproducible phylogenetic inference

We present Repro-RAxML-NG, a proof-of-concept bit-reproducible version of the widely used phylogenetic tree inference tool RAxML-NG (developed by our lab). Repro-RAxML-NG currently supports bit-reproducible tree evaluation and tree searches. In order to achieve this bit-wise reproducibility, we must ensure that our application binary does not depend on the runtime software environment. To this end, we freeze the application binary and its dynamically-linked libraries, yielding independent executions that will invoke identical CPU instructions. The current gold standard for this approach to software deployment is containerization, which is increasingly supported in HPC-environments (Boettiger, 2015). We therefore distribute Repro-RAxML-NG as a container image, which can be executed, for instance, using Docker (Merkel 2014) or CharlieCloud (Priedhorsky and Randles 2017) (rootless).

The independent evolution of distinct MSA sites constitutes a fundamental assumption of the phylogenetic likelihood model. This allows us to compute the per-site likelihoods in parallel and to subsequently reduce them to an overall likelihood score. To this end, each PE initially reduces its local site-likelihoods to an intermediate local result. Subsequently, these per-PE results are reduced into an overall global result via a parallel reduction algorithm. However, current phylogenetic tree search algorithm implementations assign genomic sites to PEs based on non-trivial load balancing algorithms—with the order and length of the assignment depending on the number of PEs (Morel *et al.* 2017). Thus, the PE-count influences the reduction order and, as a consequence, the rounding error in the overall tree score.

We alleviate this problem in Repro-RAxML-NG by decoupling the reduction order of the per-site likelihood values from the assignment of sites to PEs by the load balancer. Instead of each PE maintaining a local accumulator, Repro-RAxML-NG writes the per-site log-likelihood values into main memory, while keeping track of the global site order as defined by the input multiple sequence alignment (MSA). We then utilize a bit-reproducible parallel reduction algorithm (Section 4.2) to sum over these values by employing a calculation order that exclusively depends on the input MSA, but not on the PE-count. We apply the same principle for computing likelihood derivatives during branch length optimization. First, we compute the first and second derivatives of the per-site likelihoods and store these in main memory. Subsequently, we deploy a bit-reproducible parallel reduction algorithm to sum over all per-site values. While this does not eliminate rounding errors, it guarantees a bit-wise reproducible result by yielding a calculation order that does not depend upon the PE-count.

HPC applications also exploit SIMD instructions to substantially increase floating-point operation throughput (Dolbeau 2018). For example, the vectorized likelihood derivative kernels used in phylogenetic inference tools compute the derivatives of four distinct sites simultaneously. Different SIMD instruction sets (e.g. AVX2 instead of SSE3) provide registers and memory lanes of different widths. This may require using different CPU instructions to perform the same (mathematical) computation and thus induce distinct rounding errors. Examples include remainder loops or horizontal add instructions that do not operate across memory lanes. We alleviate some of these issues in Repro-RAxML-NG by re-implementing certain SIMD kernels and remainder loops such that they do not alter the operation order. However, the pattern compression (Stamatakis and Ludwig 2003), tip inner (distinct conditional likelihood vector functions for inner nodes that either have two tips, one tip and one inner node, or two inner nodes as children and allow for some additional optimization tricks), and site repeats (Kobert *et al.* 2017) optimizations as well as the vectorization of the likelihood derivatives that are required to optimize branch lengths (Flouri *et al.* 2015), have been disabled to keep code changes to a necessary minimum for our experiments.

We experimentally verify that Repro-RAxML-NG yields bit-identical results under varying PE-counts on the empirical datasets described in Section 6—some of which are known to cause diverging tree searches (see Supplementary material). Additionally, we verify that Repro-RAxML-NG yields bit-identical likelihoods and trees on nine distinct Intel and AMD x64 processor types, with our Docker image (see Supplementary material).

## 6 Evaluation

We conduct our experiments on the SuperMUC-NG HPC system (https://doku.lrz.de/display/PUBLIC/SuperMUC-NG). Each compute node comprises 96 GB of memory and two Intel Skylake Xeon Platinum 8174 processors with 24 cores, each running at a fixed 2.3 GHz. The nodes communicate via an OmniPath network with a fat tree topology and a bandwidth of 100 Gbit s${\;}^{-1}$. The operating system is SUSE Linux Enterprise Server 15 SP3 running Linux Kernel version 5.3.18–150300.59.63. We compile our benchmark applications using GCC version 12.2.0 with full optimizations enabled (-O3) and all assertions in RAxML-NG disabled; we employ IntelMPI 2021.9.0. Repro-RAxML-NG (https://doi.org/10.5281/zenodo.15017407) is based on RAxML-NG commit 816f9d13 (https://github.com/amkozlov/raxml-ng/commit/816f9d13), which we use as a reference.

For our runtime performance benchmarks, we utilize the same datasets previously used to evaluate the performance of RAxML-NG upon its release (Kozlov *et al.* 2019). These datasets comprise between 1371 and 21 410 970 sites, and some are thus suitable for parallel inference on dozens or hundreds of PEs.

### 6.1 Phylogenetic inference is non-reproducible under varying core-counts

We perform reproducibility experiments on 10 179 empirical phylogenetic datasets from TreeBASE (Piel *et al.* 2009), comprising more than 100 000 distinct taxa collected from over 4000 publications. These TreeBASE datasets contain 4–1912 taxa, 1–80 partitions, and 21–3 848 295 sites—with up to 207 374 unique columns in the respective MSAs. We quantify the degree of divergence of these tree searches using the Robinson–Foulds metric and the AU test (Section 4.1).

**Figure 3:**
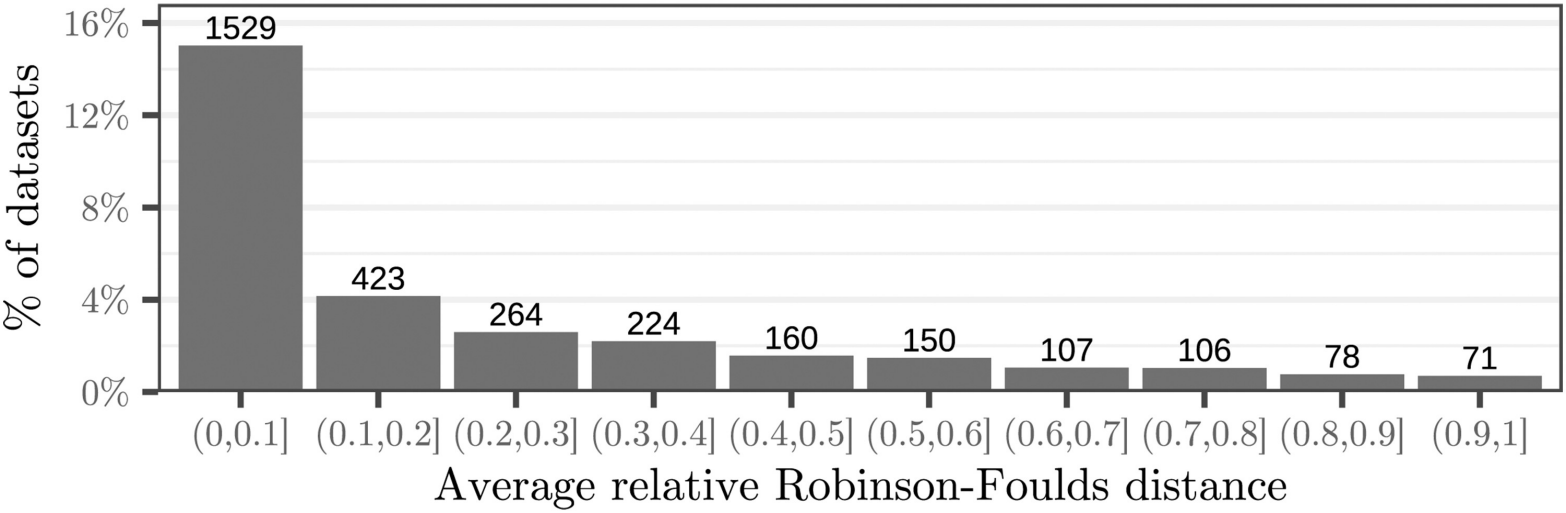
Average relative Robinson–Foulds distance (Section 4.1) between all distinct tree topologies resulting from tree searches that diverged due to different parallelization degrees. We only show datasets that yielded at least two (out of eight) distinct tree (31% of all datasets).

**Figure 4:**
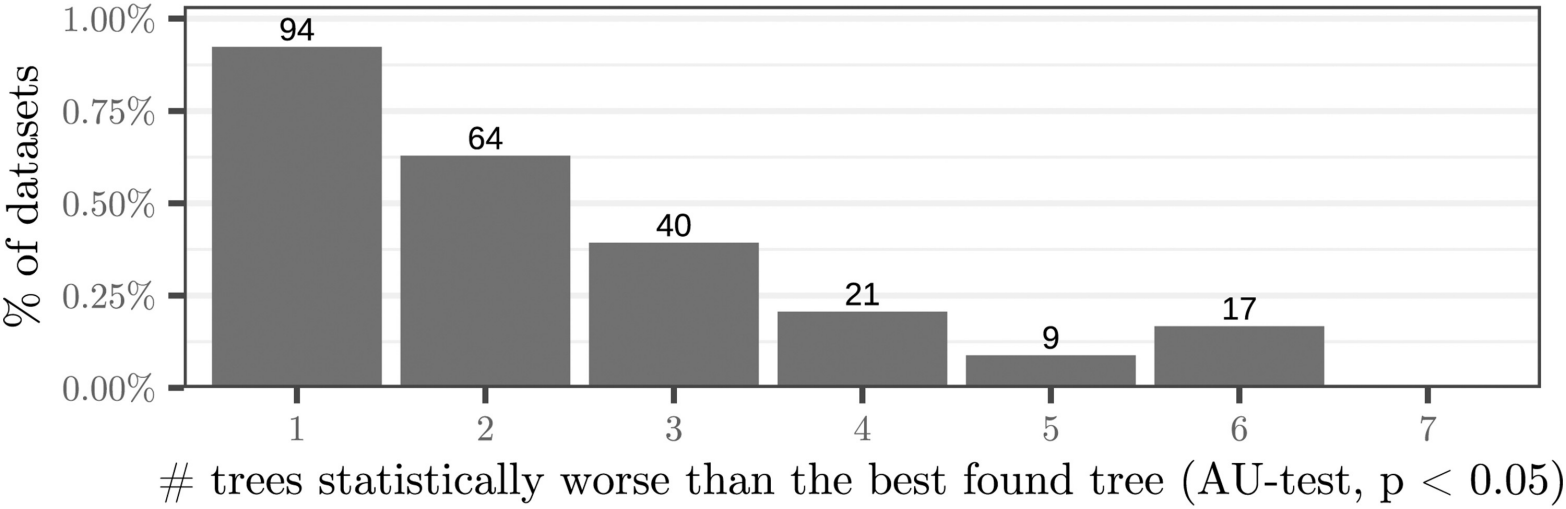
Number of trees resulting from a diverging tree search that are significantly worse than the best-known tree (AU-test, Section 4.1, *P* < .05). We only show the 8% of diverging datasets where the tree searches yielded at least one such tree.

We consider a dataset as *diverging* if the eight phylogenetic inferences conducted on it yield at least two topologically distinct trees. In our experiments, 31% (3112) datasets are diverging (Fig. 3). Further, 46% (1418) of these diverging datasets exhibit a log-likelihood difference exceeding $10^{-3}$ log-likelihood units, and 8% (245) contain significantly different trees (AU-test, *P* < .05) in the resulting tree set that comprises a total of eight trees (Fig. 4).

Compared to Shen *et al.* (2020a), who analysed 3515 single-gene datasets, we observe a higher proportion of diverging datasets (31% versus 9.3% with RAxML-NG), yet fewer significant differences (8% versus 25% of diverged datasets). This is likely due to the fact that we assess eight distinct parallelization degrees, instead of just two, as in Shen *et al.* (2020a). To prevent erroneous rejection of the correct tree in sets with highly similar likelihoods (Shimodaira 2002), we consider trees with a log-likelihood difference of less than $10^{-3}$ to not be significantly different *regardless* of the *P*-value computed by the AU-test (Shimodaira’s implementation of the AU-test generated the following warnings: “small variance”, “theory does not fit well”, and “regression degenerated”). This is in contrast to Shen *et al.* (2020a), who do not apply this (arbitrary) threshold. Without this filtering step, 38% (1186) of our diverging datasets yield significantly different trees (AU-test, *P* < .05).

In conclusion, supporting Shen *et al.* (2020b), we demonstrate that phylogenetic tree searches are sensitive to different parallelization degrees as these cause tree searches to diverge because of distinct rounding errors. This divergence introduces an unknown bias in the distribution of the resulting trees. This is orthogonal to the tree differences caused by variations in random seeds or bootstrapping (Felsenstein 1985), which are well studied (Hedges 1992). Whether differences between trees that are considered as being not significantly different from each other also have implications on their biological interpretation is non-trivial to answer and remains an open question. Therefore, we advocate for using bit-reproducible implementations of phylogenetic tree searches.

### 6.2 Runtime of isolated bit-reproducible reduction

We compare the runtime of the bit-reproducible *all-reduce* algorithms ReproBLAS (state-of-the-art), ReproRed (ours), and Gather-Bcast (recommended by the MPI-standard), as well as the non-reproducible MPI_Allreduce and Reduce-Bcast (Section 3). As only ReproRed supports reduction operators beyond summation, we compare summation performance.

As the exact data distribution impacts ReproRed’s runtime, we extract realistic data distributions from our phylogenetic inferences (Section 5). Concretely, we distribute 1000–4 110 000 double precision (64 bit) IEEE 754 floating-point elements across 48–768 PEs, maintaining an approximately equal numbers of elements per PE. We perform 4000 iterations for each data distribution, discarding the first 1000 runs for each distribution to warm up the network caches. The absolute runtimes for MPI_Allreduce range from 1.6 to 600 μs (median 10.8 μs). We verify that the reductions performed with ReproBLAS and ReproRed yield bit-identical results across different data distributions and PE-counts. Additionally, we verify that the results of all algorithms differ by less than $10^{-6}$.

**Figure 5:**
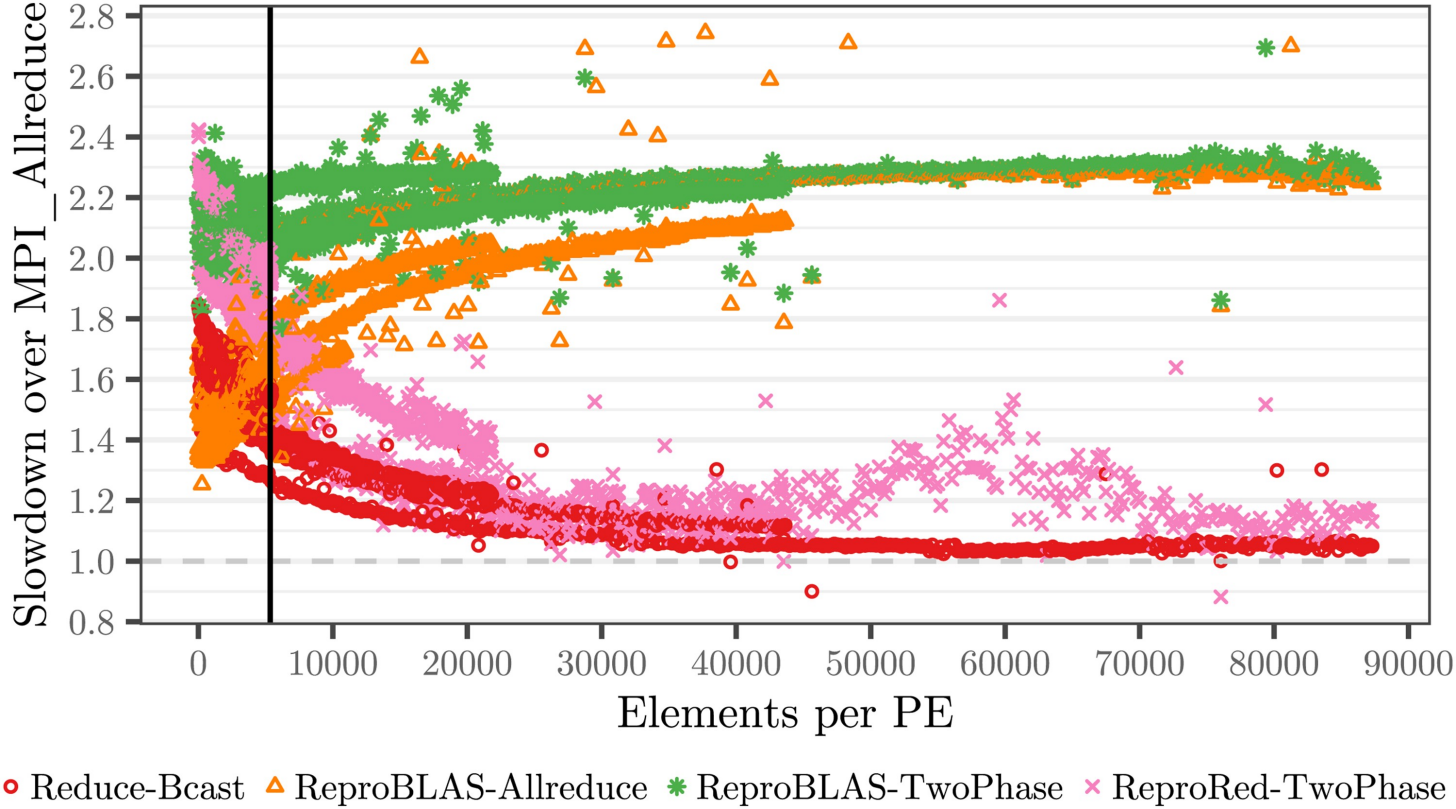
Slowdown of bit-reproducible all-reduce algorithms over a non-reproducible all-reduction. Each point represents the median runtime of 4000 iterations for the specific number of elements and number of PEs combination divided by the respective median runtime of the non-reproducible reference (MPI_Allreduce). The vertical line represents the median number of per-PE log-likelihood values in our empirical data analyses (Section 6.3).

Gather-Bcast (Section 3) is 9.8–125 (median 31) times slower than MPI_Allreduce. In addition, it requires at least one of the PEs to have sufficient memory to store *all* elements. We thus consider Gather-Bcast impractical and omit it in Fig. 5.

The ReproRed-TwoPhase *all-reduction* consists of a ReproRed reduction and a subsequent MPI_Bcast. To evaluate the overhead induced by this two-phase strategy, we measure the runtime of a non-reproducible local reduction followed by an MPI_Reduce and an MPI_Bcast (*Reduce-Bcast*; Fig. 5). The median number of elements per PE in our phylogenetic experiments is 1935, yielding Reduce-Bcast 61% slower (median) than all-reduce. However, for more than 45 000 elements per PE, Reduce-Bcast is merely 5% slower (median).

We find that ReproRed outperforms ReproBLAS-TwoPhase under most data distributions exceeding 3000 elements per PE, and outperforms ReproBLAS-Allreduce exceeding 10 000 elements per PE (Fig. 5). Both, ReproRed and ReproBLAS exchange only the theoretical minimum number of messages for a reduction. While ReproRed performs less local work than ReproBLAS, it also increases the number of computations along the critical path (Supplementary material). In accordance to this, we observe that ReproRed is slower than ReproBLAS for less than a few thousand elements per PE. However, as the number of elements per PE increases, local computation starts compensating these effects. This explains ReproRed’s notably faster performance on data distributions with tens of thousands of elements per PE (Fig. 5). For instance, ReproRed is 37% (median) times faster than ReproBLAS on 8000–12 000 elements per PE and 92% (median) times faster on more than 30 000 elements. Note, however, that ReproBLAS utilizes additional local work to guarantee the final result accuracy. In contrast, ReproRed’s pair-wise summation merely increases the accuracy (Higham 1993). Further, ReproBLAS is restricted to summation as reduction operator, while ReproRed supports arbitrary associative reduction operators.

### 6.3 Bit-reproducible phylogenetic tree search

**Figure 6:**
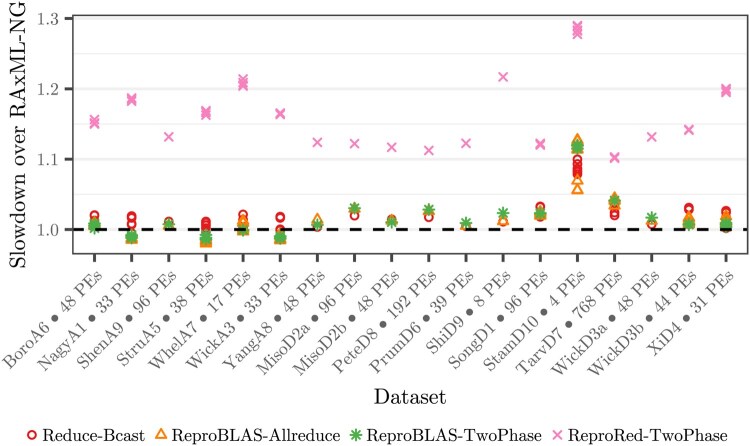
Slowdown of bit-reproducible Repro-RAxML-NG over reference RAxML-NG. Reduce-Bcast uses a non-reproducible reduction but includes the remaining modifications required for bit-reproducibility. The variants ReproBLAS and ReproRed yield bit-reproducible results.

We assess the slowdown of our bit-reproducible Repro-RAxML-NG (Section 5) in comparison to the unmodified reference RAxML-NG (Fig. 6). Specifically, we evaluate Repro-RAxML-NG with the reproducible all-reduce algorithms ReproBLAS (state-of-the-art), Gather-Bcast (recommended by the MPI-standard), and ReproRed (ours). Further, we present results for the non-reproducible MPI_Allreduce and TwoPhase approaches. We use the PE-counts recommended by RAxML-NG’s --parse for the respective datasets, resulting in 253–11 903 (median 5569) unique MSA columns per PE and 1935 (median) all-reduce operations per second (for the RAxML-NG reference version). For consistency, we disable optimization features not supporting bit-reproducibility in all variants (Section 5). We exclude datasets where the tree searches diverged, as this yields the runtimes not comparable and verify that the resulting log-likelihood scores of trees differ by less than $10^{-3}$ log-likelihood units among all variants.

Repro-RAxML-NG using the Gather-Bcast all-reduce algorithm is 21%–386% (median 26%) slower than the reference. It is consistently the slowest variant across all configurations; we hence omit it from further discussion.

Repro-RAxML-NG ReproRed-TwoPhase is 10%–29% (median 16%) slower than the reference. However, when employing the bit-reproducible ReproBLAS all-reduction, reproducibility induces only 0%–6.7% (median 0.9%) slowdown. This is consistent with the isolated measurements (Section 6.2), where ReproBLAS outperforms ReproRed-TwoPhase on less than 10 000 elements per PE. Even though some of RAxML-NG’s optimizations are disabled, due to lack of bit-reproducible implementations (Section 5), we are cautiously optimistic that implementing bit-reproducible phylogenetic tree inference tools is feasible.

## 7 Reproducible Bayesian phylogenetic inference

Bayesian inference (BI) methods such as RevBayes (Höhna *et al.* 2016), and BEAST (Bouckaert *et al.* 2019) face the same fundamental reproducibility challenge as ML methods. This is because the per-site log-likelihoods also need to be accumulated to obtain a single tree score. Depending on the parallelization mode, RevBayes and BEAST either implement this reduction either via a non-reproducible reduction algorithm or via reproducible, yet less scalable parallelization schemes (e.g. using only one PE per MSA partition, sequential accumulation of the per-site likelihoods, etc). In contrast to ML, BI methods can also execute multiple Markov chains in parallel (e.g. independent runs or cold versus hot chains). Therefore, they rely on per-site level parallelism to a lesser extent than ML to attain scalability. Thus, we expect the performance penalty induced by bit-reproducible reductions to be less pronounced than for ML methods. To which extent reduction-induced numerical deviations affect the composition of the posterior tree sample obtained via BI remains to be investigated. The effects might be less pronounced as BI methods do not strive to yield a point estimate such as ML. In other words, numerical deviations could potentially be “averaged out” by the MCMC mechanism.

## 8 Conclusion and future work

We highlight the issue of non-reproducibility in phylogenetic tree inference and present the first (proof-of-concept) bit-reproducible phylogenetic tree inference tool. We consider this an important step toward bit-reproducible—and thus more reliable—phylogenetic tree inferences. We also develop the first open-source bit-reproducible reduction algorithm supporting associative reduction operators, which we already integrated into the open-source MPI-wrapper library KaMPIng (Uhl *et al.* 2024) to facilitate the transition to bit-reproducible code.

In future work, we aim to develop bit-reproducible kernels for calculating the derivatives of the phylogenetic likelihood and to port optimizations like pattern compression (Stamatakis and Ludwig 2003) to Repro-RAxML-NG. We also intend to implement a recursive doubling all-reduce variant of ReproRed (see Supplementary material), to further accelerate all-reduction. Additionally, we plan to extend ReproRed to support both, inclusive, and exclusive prefix sums.

## Supplementary Material

btag044_Supplementary_Data

## Data Availability

The data underlying this article are available in Zenodo, at https://doi.org/10.5281/zenodo.15592496.
